# A Quarter of a Century of Evidence-Based Severe Trauma and Orthopaedic Injury Management

**DOI:** 10.3390/jcm15145565

**Published:** 2026-07-15

**Authors:** Dirk Stengel, Axel Ekkernkamp, Paul A. Grützner

**Affiliations:** 1 Research Methods and Information Management, Business Division Medicine, BG Kliniken—Klinikverbund der gesetzlichen Unfallversicherung gGmbH, Leipziger Platz 1, 10117 Berlin, Germany; 2 BG Klinikum Unfallkrankenhaus Berlin gGmbH, Warener Str. 7, 12683 Berlin, Germany; axel.ekkernkamp@gmx.de; 3 Business Division Medicine, BG Kliniken—Klinikverbund der gesetzlichen Unfallversicherung gGmbH, Leipziger Platz 1, 10117 Berlin, Germany; paul.gruetzner@bgu-ludwigshafen.de; 4 BG Klinik Ludwigshafen, Ludwig-Guttmann-Straße 13, 67071 Ludwigshafen am Rhein, Germany

We, the Guest Editors of this Special Issue, and all its contributors, do not want to annoy or bore you, our respected audience, with another elaboration on the term and principles of evidence-based medicine [1] and its necessary modifications to suit the various surgical disciplines. An abundance of articles has been published in the surgical literature on this matter since its emergence in the early 2000s [2,3,4]. “Everything has already been said, just not by everyone” is a famous quote by the Munich comedian, cabaret artist, and author Karl Valentin (1882–1948). This timeless quote is used to comment on superfluous repetitions in debates. Although simplistic in many ways, the assumption that accidents do not just happen, but are caused, illustrates the importance of injury prevention at the population level [5]. For the individual, however, they remain unexpected and unpredictable events with consequences ranging from a short period of sick leave and minor pain to life-threatening conditions, weeks of intensive care, and life-long disability. It is the responsibility of interprofessional trauma care teams to be prepared for acute situations in which shared decision-making is limited or impossible, and to provide the best possible care by combining clinical (and surgical manual) expertise with the best available scientific evidence for our patients. Some of the scientifically funded milestones achieved over the past 25 years are compiled in this Special Issue, summarised by renowned experts in their respective fields. We would like to highlight three topics here that exemplify how evidence from translational research has fundamentally changed clinical practice, patient experiences, and outcomes.

Severe burns are among the most devastating, painful, and mutilating injuries imaginable [6]. In-depth exploration of system pathophysiology, individualised countermeasures across different phases of care, and regenerative medicine enabled a shift in focus from pure survival to outcomes that maximise health-related quality of life, function, and participation—all based on scientific data [7].

Complex knee injuries, including multi-ligament tears [8] and fractures of the distal femur [9] and proximal tibia [10], bear a high risk of immobilisation. Clinical research has shown that biological, minimally invasive approaches to reconstruct and replace soft tissue structures, including locking plate technology for fracture fixation and restoration of anatomy, assisted by intra-operative three-dimensional imaging, navigation, and robotics, are likely to improve functional outcomes and should be offered in a joint discussion between providers and patients [11,12].

The management of thoracolumbar spine fractures likewise benefited from the emergence of intraoperative 3D imaging, navigation and minimally invasive approaches [13]. In industrialised countries, no spine unit should be equipped without this technology [14].

Many other examples of how disruptive technology changed structural and process quality to enhance the goal of healthcare, namely, quality of outcomes for patients and their relatives, are contained in this Special Issue, and we are grateful to all author teams who accepted the challenge of condensing years of clinical and scientific work. We could not elaborate on the importance of establishing national trauma systems and networks [15], standardised work-up algorithms [16], and certified high-volume trauma centres [17] in improving outcomes. A recent Cochrane Review reminds us that much research remains to be conducted to prove that we are on the right track [18]. We also do not want to mention artificial intelligence (AI) applications here—you will find them in this Special Issue.

Wait—we forgot something of utmost importance. Evidence-based surgical practice is impossible without barrier-free access to the scientific literature. Open access publishing established itself as a sustainable model. Access to information has become much easier, but the information load itself is exploding exponentially, making it increasingly difficult to separate the wheat from the chaff. Distinct methodological expertise with a clinical eye [19] is required to find the needle in the haystack—to reveal both overenthusiastic data interpretation and inconsistencies [20]. Besides the obvious need for multicentre, international trials to study the added value and utility of proposed novel interventions compared with the standard of care, outstanding individual cases may prompt systematic research and change the way we treat patients in the future. Without real-world information, we cannot formulate reasonable research hypotheses, or determine whether experimental results translate into differences and patient advantages in daily practice. Please enjoy reading this Special Issue!
